# Spatial Distribution Characteristics of Suitable Planting Areas for *Pyrus* Species under Climate Change in China

**DOI:** 10.3390/plants12071559

**Published:** 2023-04-05

**Authors:** Mi Wang, Zhuowei Hu, Yongcai Wang, Wenji Zhao

**Affiliations:** College of Resources Environment and Tourism, Capital Normal University, Beijing 100048, China

**Keywords:** *Pyrus*, climate change, CMIP6, Maxent, planting distribution

## Abstract

Planting suitability determines the distribution and yield of crops in a given region which can be greatly affected by climate change. In recent years, many studies have shown that carbon dioxide fertilization effects increase the productivity of temperate deciduous fruit trees under a changing climate, but the potential risks to fruit tree planting caused by a reduction in suitable planting areas are rarely reported. In this study, Maxent was first used to investigate the spatial distribution of five *Pyrus* species in China, and the consistency between the actual production area and the modeled climatically suitable area under the current climatic conditions were determined. In addition, based on Coupled Model Intercomparison Project Phase 6, three climate models were used to simulate the change in suitable area and the migration trend for different species under different emission scenarios (SSP1-2.6, SSP2-4.5, SSP3-7.0 and SSP5-8.5). The results showed that the suitable area for pear was highly consistent with the actual main production area under current climate conditions. The potential planting areas of *P. ussuriensis* showed a downward trend under all emission paths from 2020 to 2100; other species showed a trend of increasing first and then decreasing or slowing down and this growth effect was the most obvious in 2020–2040. Except for *P. pashia*, other species showed a migration trend toward a high latitude, and the trend was more prominent under the high emission path. Our results emphasize the response difference between species to climate change, and the method of consistency analysis between suitable planting area and actual production regions cannot only evaluate the potential planting risk but also provide a reasonable idea for the accuracy test of the modeled results. This work has certain guiding and reference significance for the protection of pear germplasm resources and the prediction of yield.

## 1. Introduction

The climate plays a key role in defining the geographic range of plants, and climate change is expected to severely influence plant distributions in the forthcoming decades [1,2]. As a perennial crop that is affected by the climate throughout the year, fruit trees are considered to be vulnerable to climate change [3]. A temperature rise due to climate change leads to an advance in the spring phenology of fruit trees in high-altitude areas of Switzerland, which increases the risk of frost [4], and the amount of winter cold required for temperate trees to overcome dormancy is expected to be greatly reduced in the future in the most deciduous fruit-growing areas [5]. In this context, determining how species and cultivars adapt to certain climates is of primary interest in research and decision-making, to explore the potential for cultivation in new areas and to allow for the development of adaptation strategies to climate change in current locations.

The pear (*Pyrus communis* L.) belongs to the rosaceous family, and is a typical fruit of temperate regions. It is the fifth most widely produced fruit in the world, being produced mainly in China, Europe and the United States [6]. Among them, the pear industry has become the third largest fruit industry following the apple and citrus industries in China [7]. As the largest producer and exporter of pears, China accounted for about 71%, 69% and 18% of pear output, cultivation area and export volume of the world, respectively, in 2020 [8].

The growing range of plant species and cultivar adaptation to a given area is strongly influenced by the climate, and, in particular, by temperature and moisture regimes. The annual average surface temperature in China rose at a rate of 0.26 °C/10 years, which was significantly higher than the global average (0.15 °C/10 years) during the same period from 1951 to 2020 [9]. The average annual precipitation in China shows an increasing trend, and the regional precipitation differences are prominent, whereby among which the northwest region has had the most significant increase in precipitation [10]. Studies have shown that with the further intensification of climate warming, the phenological period of *Pyrus* *bretschneideri* in Shaanxi Province has tended to be an early phenological period in spring and delayed phenological period in autumn [11,12], showing that with climate warming, the climate-suitable area of *Pyrus xerophila* will be further reduced in the future. Many studies have focused on the impact of climate change on pear phenology or on a single climatic factor on its yield. However, it is still not clear whether *Pyrus* are suitable for planting promotion outside their main production areas, and how the current pear-suitable planting areas will change in the future.

A suitable species distribution model (SDM) is often required to assess the potential impact of climate change on spatial distribution [13]. The SDMs refers to the method of obtaining the relationship between the distribution sample information of the species and the corresponding environmental information, and applying this relationship to a specific area to realize the prediction of the geographic distribution of the target species [14]. It has become an important tool for studying the interaction between the spatial pattern of the geographical distribution of the species and the environment under global climate change [15,16]. The most common SDMs were developed by using the genetic algorithm for rule-set prediction (GARP) [17], CLIMEX [18], random forest (RF) [19] and maximum entropy (Maxent) [20], etc. However, Maxent has higher prediction accuracy [21,22] and is widely used in the prediction of potential climate-suitable areas of species, the prediction of the invasion trend of alien species, biodiversity conservation and endangered species conservation [23,24,25].

Although Maxent is a conventional SDM, overlaying the model output with yield statistics and calculating their consistency provides a new way to study the potential impact of climate change on the suitability distribution of fruit tree. In this study, we selected five widely cultivated species *Pyrus bretschneideri, Pyrus pyrifolia, Pyrus ussuriensis, Pyrus sinkiangensis* and *Pyrus pashia* to be models. The objectives of this study are to (1) determine the dominant environmental variables that affect the differences in the potential planting regions of different species; (2) identify the suitable distribution areas of representative pear species under current climatic conditions using Maxent and evaluate its consistency with the actual production area; (3) project the centroid shift trend of different species and the area change in potential planting regions under different emission paths and (4) assess potential planting risks from consistency changes in future climatic conditions.

## 2. Results

### 2.1. Model Verification

**Figure 1:**
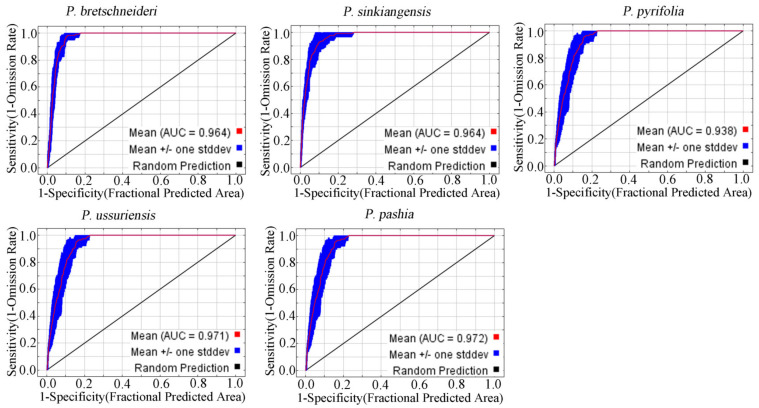
Maxent model accuracy verification diagram of five *Pyrus* species.

Figure 1 shows that the average AUC of the Maxent model is greater than 0.9 after being run for ten times, and among which *P. bretschneideri* is 0.964, *P. ussuriensis* is 0.971, *P. sinkiangensis* is 0.963, *P. pashia* is 0.972 and *P. pyrifolia* is 0.938. The results suggest that the model’s prediction accuracy could be rated as showing excellent performance. This model can therefore be used to identify the suitability habitats for *Pyrus* in China.

### 2.2. Percentage Contribution of Environmental Variables and Response Curve

**Figure 2:**
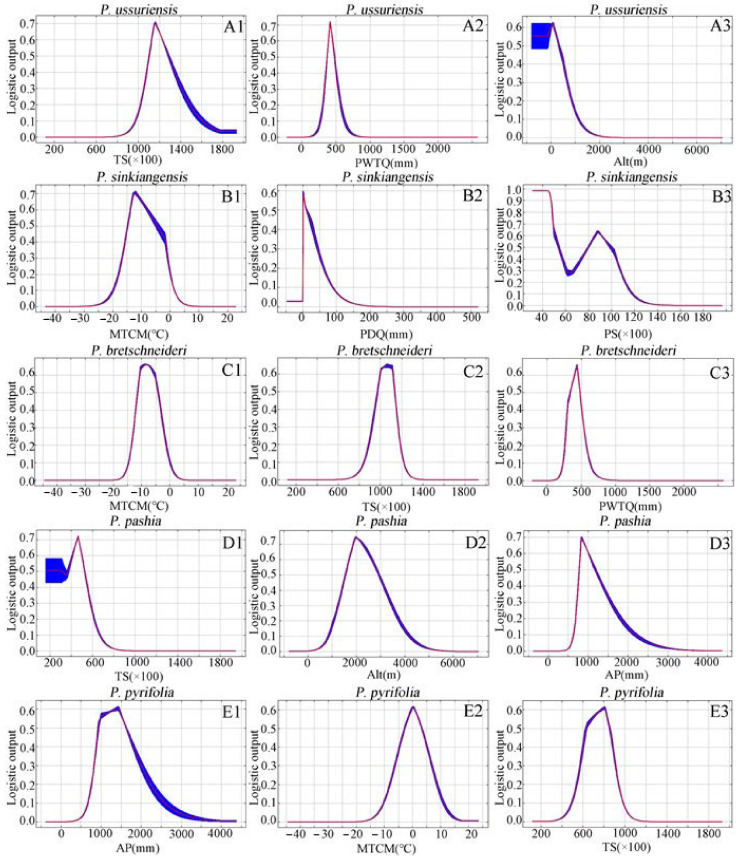
Response curves of the existence probability (habitat suitability) of five species of pear to major environmental variables with simulated distribution under current climatic conditions. (**A1**–**A3**) *P. ussuriensis*, (**B1**–**B3**) *P. sinkiangensis*, (**C1**–**C3**) *P. bretschneideri*, (**D1**–**D3**) *P. pashia*, (**E1**–**E3**) *P. pyrifolia*. The Maxent logistic output (also known as habitat suitability) was represented by the vertical axis, while the environmental variables were represented by the horizontal axis. With a logical output value greater than 0.5, the probability of species presence under the given condition was considered to be suitable for the species. Note: the red curves show the average over 10 replicate runs; blue bands show the standard deviation (SD) calculated over 10 replicates.

The percentage contribution values of the three identified factors averaged over 10 replicate runs are shown in Table 1. The results reveal that the min. temperature for the coldest month (MTCM) is a dominant variable that affects the growing suitability for all five *Pyrus* species. The other dominant factors are different for different species. For example, temperature seasonality (TS), min. temperature for coldest month (MTCM) and altitude (ALT) (Figure 2A1–A3) account for 77.3% of variation for *P. ussuriensis*. For *P. sinkiangensis,* min. temperature for coldest month (MTCM), precipitation of driest quarter (PDQ) and precipitation seasonality (PS) (Figure 2B1–B3) are the dominant factors, while for *P. bretschneideri*, the three factors were min. temperature for coldest month (MTCM), temperature seasonality (TS) and precipitation of wettest quarter (PWTQ) (Figure 2C1–C3), accounting for 74.7% of variation. The three most dominant variables for *P. pashia* included temperature seasonality (TS), altitude (AIL) and annual precipitation (AP) (Figure 2D1–D3), accounting for 86.9% of variation. The three most dominant variables for *P. pyrifolia* included annual precipitation (AP), min. temperature for coldest month (MTCM) and temperature seasonality (TS) (Figure 2E1–E3), accounting for 88.1% of variation.

The response curves of the three environmental variables with the highest contribution rate among different species were extracted for analysis (Figure 2), which shows how the logical prediction of the Maxent model changes with the changes in each environmental variable.

The results showed the following: the optimal TS value of *P. ussuriensis* is 1100–1300. TS is the ratio of the standard deviation of the monthly average temperature to the average value of the monthly average temperature (×100). It is a measure of the temperature change in a year. The greater the seasonal variation coefficient, the greater the temperature change. For *P. ussuriensis*, the precipitation of the wettest quarter in the highly suitable area is below 500 mm and the highly suitable areas are under 300 m above sea level. The suitable area for the growth of *P. sinkiangensis* is the area where the MTCM is −15 °C to 8 °C; in addition, the precipitation of *P. sinkiangensis* in the driest season is less than 20 mm, and its PS in different planting areas is quite different. When the MTCM value is −11 °C to −4 °C, *P. bretschneideri* show higher growth suitability, with the best value ranging between 950 and 1120, and the PWTQ ranging between 300 and 500 mm. According to the response curve of TS, *P. pashia* grows in areas with a very low seasonal temperature change. The annual precipitation in its best suitable area is between 700 mm and 1300 mm, and the highest value is 800 mm. Different from the altitude of other species, *P. pashia* is mainly distributed in plateau areas between 1500 m and 3000 m above sea level with small seasonal changes in temperature. When AP is in the range of 500 mm–1400 mm, the habitat suitability and AP are highly positively correlated. Additionally, the MTCM for *P. pyrifolia* ranged from −4 to 4 °C.

### 2.3. Suitable Planting Regions of Pyrus under Current Conditions

**Figure 3:**
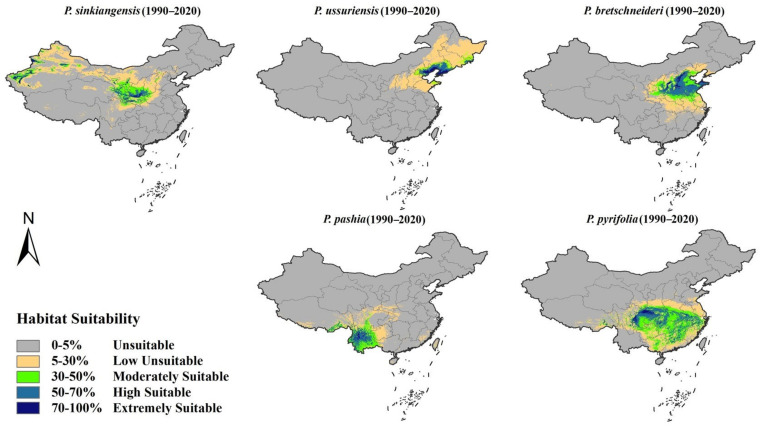
Suitable planting regions for different species of *Pyrus* under current climate conditions (1990–2020).

The suitable planting range for five species of *Pyrus* under the present climate (1990–2020) is presented (Figure 3) based on observed occurrences and current environmental variables forecasted by Maxent. As shown in Figure 3, the highly and extremely suitable areas of *P. ussuriensis* are mainly distributed in northern Hebei and southwestern Liaoning; the moderately suitable and low-suitability areas are mainly distributed in Jilin, Heilongjiang, Inner Mongolia and other places; the highly suitable areas of *P. bretschneideri* are mainly distributed in central and southern Hebei, central and southern Shanxi, Shandong, Anhui and northern Jiangsu; the moderately suitable and low-suitability areas are widely distributed in the eastern provinces; the highly suitable areas of *P. sinkiangensis* are mainly distributed in the Kashgar, Korla, Aksu and Turpan basins and some areas of Gansu, Ningxia and Shaanxi; the moderately suitable and low-suitability areas are mainly distributed in Gansu and Shaanxi; the highly suitable areas of *P. pashia* are mainly distributed in the middle of Yunnan Province and the valley of southern Tibet; the moderately suitable and low-suitability areas are widely distributed in Yunnan, Guizhou and some areas of Sichuan; the highly suitable areas of *P. pyrifolia* are mainly distributed in the northeast and middle of Sichuan and parts of Jiangxi and Zhejiang and the moderately suitable areas are widely distributed in the Yangtze River Basin.

### 2.4. Consistency between the Potential Planting Regions and the Actual Main Production Counties under Current Climate Conditions

**Figure 4:**
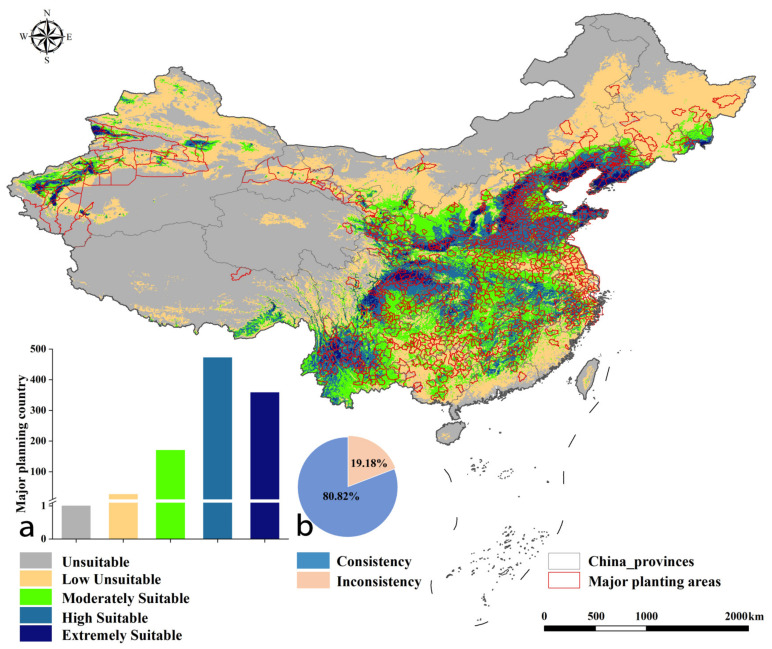
Consistency map of suitable regions and main production counties under current climatic conditions. Note: the bar chart (**a**) shows the number of main pear-producing counties located in different areas of habitat suitability, and the pie chart (**b**) shows the proportion of consistency.

As shown in Figure 4, the classification map of suitable areas and the vector map of the main pear-producing areas were superimposed and analyzed; then, the consistency between the main pear-producing counties and the potentially suitable areas was calculated. The results showed that there were 472 and 358 counties with pear production located in the highly suitable areas and the extremely suitable areas, respectively. The high consistency between the main producing areas and the potentially suitable areas accounts for 80.82% under current climatic conditions, which further confirms the reliability of the model results.

### 2.5. Future Changes in Potential Planting Areas for Pyrus of Different Species

**Figure 5:**
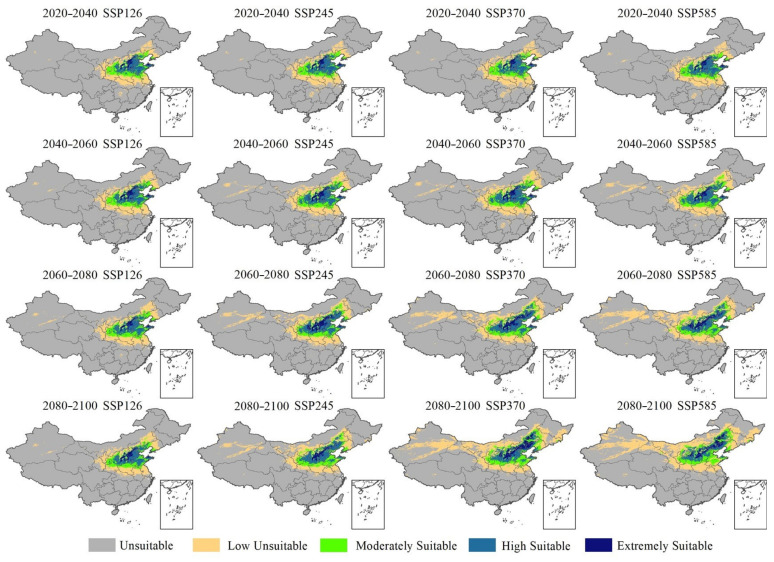
Distribution map of climatically suitable areas of *P. bretschneideri* under future climatic conditions in China.

We took the average of the prediction results of the three GCM models to create a climate-suitable area distribution map, which was used to reduce the uncertainty of choosing a single GCM simulation result [26,27]. Figure 5 shows the changes in the suitable climate zone for *P. bretschneideri* under different emission paths (SSP1-2.6, SSP2-4.5, SSP3-7.0 and SSP5-8.5) in the future. (The distribution maps of the other four species are shown in Figures S2–S5.)

**Figure 6:**
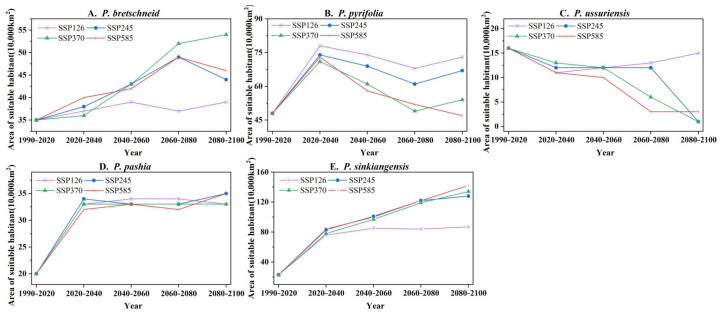
Change map of potential planting areas (habitat suitability > 0.5) for different species of (**A**) *P.bretschneld*, (**B**) *P. pyrifolia*, (**C**) *P.ussuriensis*, (**D**) *P.pashia*, (**E**) *P.sinkiangensis*. (i.e., SSP1-2.6, SSP2-4.5, SSP3-7.0 and SSP5-8.5 have been modeled from 2020 to 2100, respectively).

In order to generate the presence/absence map of *Pyrus*, the continuous probability values generated by Maxent were converted into a threshold-based binary prediction map [28]. We chose 0.5 as the threshold. The part of the prediction result above the threshold indicates higher habitat suitability (hereinafter referred to as suitable planting regions); the part below the threshold indicates low habitat suitability (hereinafter referred to as unsuitable planting regions). Under the current climate conditions, the area of the suitable planting regions in descending order is *P. pyrifolia* (48 × 10^5^ km^2^), *P. bretschneideri* (35 × 10^5^ km^2^), *P. sinkiangensis* (23 × 10^5^ km^2^), *P. pashia* (20 × 10^5^ km^2^) and *P. ussuriensis* (16 × 10^5^ km^2^). *P. ussuriensis*’s suitable planting area will show different degrees of reduction under different emission paths in the future. The suitable planting areas for the other four species show different degrees of increase from 2020 to 2040 and they show a trend of slowing and declining growth from 2040 to 2100. Among them, *P. sinkiangensis* have the largest increase (Figure 6).

**Figure 7:**
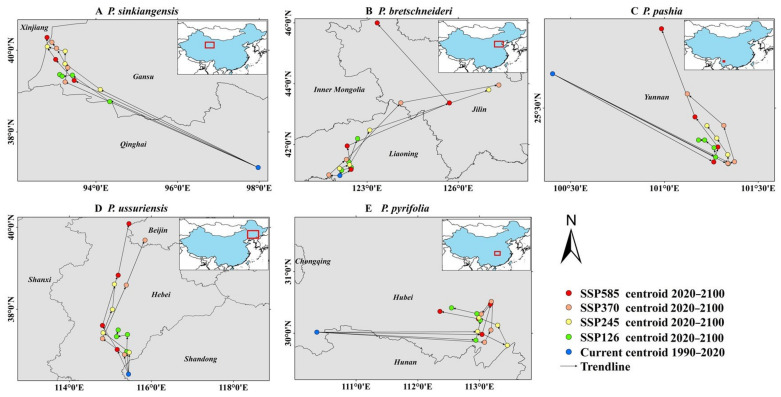
The centroid shift map of the climate-suitable areas for different species of (**A**) *P.sinkiangensis*, (**B**) *P.bretschneideri*, (**C**) *P.pashia*, (**D**) *P.ussuriensis* and (**E**) *P. pyrifolia*.

We used geographic distribution measurement tools to fit the distribution range of the species to a single centroid (center) point and create a vector file to predict the migration trend of a species by tracking the changes in the centroid in different periods [29]. We drew the centroid shift map of potentially suitable areas for *Pyrus* of five species under different emission paths (SSP1.2-6, SSP2.4-5, SSP3.7-0 and SSP5.8-5) in the future (Figure 7), which was used to assess the potential impact of different species migration trends on the consistency in the suitable climate regions and the actual planting areas.

The distribution centroid for the five species is as follows: *P. bretschneideri* (115.44, 36.42), *P. ussuriensis* (122.11, 40.98), *P. sinkiangensis* (97.97, 37.13), *P. pashia* (100.40, 25.68) and *P. pyrifolia* (110.36, 30.02). In the future, there will be no obvious migration trend in the suitable regions of *P. pashia*, showing high stability (Figure 7C), and the suitable regions of other species of *Pyrus* will show obvious different degrees of northward migration trends (Figure 7A,B,D,E). Therefore, with different degrees of migration from climate-suitable areas, the consistency between the main pear-producing regions and suitable areas of *P. ussuriensis* has a higher risk reduction. Additionally, by comparing the area change and the centroid shift trends of the suitable regions under different emission paths, we find that the area change and centroid shift trends of each species are more obvious under the high emission path. Therefore, the consistency in the suitable regions and the actual main pear-producing areas under the high-emission path faces a higher risk.

## 3. Discussion

### 3.1. Differences in the Impact of Environmental Variables on Different Species of Pyrus

Through the analysis of environmental variables with high contribution rates on the suitability distribution of different pears, we found that the main temperature factors affecting the spatial suitability of *Pyrus* were MTCM and TS, and the main precipitation factors were AP, PWTQ and PDQ. These identified factors are consistent with previous studies of [12,30], whereby the authors reported that seasonal variation in temperature and annual precipitation were the main environmental variables affecting the spatial distribution of some species of *Pyrus*. Different species show great differences in the lowest temperature of the coldest month [31], and other studies have shown that the pear most resistant to low temperatures is *P. ussuriensis*, which can withstand the extreme low temperature of −45 °C. *P. sinkiangensis* have strong resistance to cold and drought, followed by *P. bretschneideri*, *P. pashia* and *P. pyrifolia* which have low cold resistance. A low temperature was one of the main factors affecting the distribution of all pear species [32]. Due to the difference in environments and the adaptation of pears, other factors that affect the distribution of suitable planting areas are different for different species. The average altitude is the key environmental factor for *P. pashia*, *P. sinkiangensis* and *P. ussuriensis*, which are widely distributed in mountains below 3500 m [33]. Of the 12 variables used for the model establishment, soil variables all had lower contribution rates than others, and related studies have also shown that as the spatial range of the environmental factor grid used in the analysis increases, the importance of soil declines [13]. Identifying the dominant environmental variables that affect distribution of representative species provides insight into understanding the effects of changes in environments on the suitability of species in a certain area.

### 3.2. Changes in the Distribution Range of Pyrus and Climatic Risks of Planting

Our modeled results showed that unlike the other four suitable species which obviously migrate to high latitudes (Figure 7), *P. pashia* will not show significant species migration under any emission paths in the future, showing a high degree of stability. Relevant studies have shown that species have a tendency to migrate to high latitudes under the changing climate [34], and a high altitude can largely limit the migration of species [35]. Considering that *P. pashia* is mainly distributed in the Yunnan–Guizhou Plateau and the average altitude of growth is significantly higher than that of other species [33], altitude is likely to be the main reason that its migration trend is significantly different from the other species. Therefore, our results further confirm that high altitude can restrict species migration to a certain extent. In view of the expansion of the distribution area and the stable centroid of the climatic suitability of sand pears, the pears in this area will have high planting potential in the future.

The climatically suitable areas of *P. ussuriensis* have been reduced under different emission paths, and the centroids of distribution all show a large shift to the high latitudes. Research has shown that fruit trees in temperate and subtropical regions have higher requirements for the length of low temperature to end the dormancy period of branches and buds, and future increases in climate temperature may lead to insufficient low temperature time for fruit trees in some areas, thereby affecting the survival suitability of fruit trees [36]. Northeast China where *P. ussuriensis* is grown is one of the most significant regions in China in responding to climate warming, and continued climate warming may not be able to meet the low temperature accumulation of *P. ussuriensis* growth in the future, resulting in the shrinking of the climatically suitable areas and a northward shift. Zhou [37] showed that although national pear production has increased due to an improvement in agronomic management, the planting area of pears first increased and then decreased in Northeast China over the past 40 years, and the proportion of pear production has declined more, which climate change has contributed to. Future climate change may further reduce the suitable planting area of pears in this region. In order to stabilize pear yields, new species need to be introduced, such as *P. sinkiangensis*, which also have good climate suitability in this region. In contrast, the suitable planting areas for *P. bretschneideri*, *P. pyrifolia* and *P. siniangensis* will be further expanded and their distribution centroids will migrate to higher latitudes under different emission pathways in the future. Zhou [37] reported that the national proportion of pear yield in Northwest China and the Yangtze River Basin has been increasing in the past 20 years. Especially in Xinjiang, with the expansion of planting areas, the national proportion of pear yield has expanded from 2 to 9% [38]. Therefore, in view of the differences in the response of the five pears to climate change, we emphasize the development of different planting and management policies to deal with potential climatic risks of planting.

### 3.3. The Guiding Role of Species Distribution Models in Planting Management of Pyrus

Based on the modeled results, the suitable planting range of different pears was determined and divided into five grades. The results (Figure 3) show that the potential suitable planting regions of *Pyrus* include some areas with relatively little planting promotion efforts, such as parts of Tibet, Qinghai and Inner Mongolia province. These areas can improve the yield by introducing corresponding planting varieties. In addition, our findings suggest that there is a certain overlap between the climatically suitable zones for different species, and new planted varieties can first be promoted in these areas. For example, not only *P. sinkiangensis* but also *P. bretschneideri* have high climatic suitability in the northwest region. In addition to the Yangtze River Basin, *P. pyrifolia* can also be considered for planting in southern Tibet such as in Linzhi and Cangdu. Therefore, the delimitation of suitable regions has important practical significance for the utilization of the advantages of location resources during the planting process and the increase in pear farmers’ ability to adapt to climate change.

Furthermore, fruit trees are falling into the dilemma of biodiversity reduction under the long-term impact of global warming. Some varieties that cannot better adapt to changes are facing a serious threat to genetic resources [39]. Studies have shown that mountainous areas retain higher species richness and biodiversity in the process of long-term climate change [40]. Therefore, while introducing other planting varieties and expanding the planting area, methods such as establishing a germplasm resource bank or ex situ protection are adopted to minimize the adverse effects of human variables and climate change on local pear varieties in high-altitude areas such as Yunnan.

### 3.4. Uncertainty and Limitation

The first limitation is the uncertainty of the model itself, including the selection of sample distribution points and input variables during modeling. Because land use changes are greatly disturbed by human variables, the analysis of suitability distribution areas and distribution centroid shift trends is based on environmental variables and land use was not considered. Based on the interference of human variables, it is necessary to combine the current status and change trends of land use in different regions to make more reasonable use of our research results.

Many studies have reported the importance of considering the effects of pest and diseases on plant production under climate change [41,42], and the probability of occurrence of pests and diseases in China caused by climate change in the future would be greater and would affect the yield and ecological suitability of fruit trees [43,44]. However, the current model cannot simulate the impact of increased pests and diseases caused by climate change on the habit suitability of fruit trees. This is also a key direction for studying the impact of climate change on fruit trees in the future.

Nevertheless, our results provide insight into what the spatial distribution of a suitable pear area would look like in China under a changing climate.

## 4. Materials and Methods

### 4.1. Data Description

#### 4.1.1. Species Distribution Data and Preprocessing

China includes a cold temperate zone, a middle temperate zone, a warm temperate zone, a plateau climate zone, a subtropical zone and a tropical zone [45]. The *Pyrus* species are mainly distributed in the temperate zone and subtropical zone [46,47]. The main pear-producing areas can be divided into the warm-temperate western *P. sinkiangensis* planting areas (Xinjiang, Gansu, Ningxia, Qinghai and Shaanxi province); the warm-temperate eastern *P. bretschneideri* planting areas (Shanxi, Hebei, Shandong, Henan, Anhui and Jiangsu province); the *P. ussuriensis* planting area in the mid-temperate zone (North Hebei, Beijing, Tianjin, Jilin and Liaoning province); the *P. pyrifolia* planting areas in the subtropical Yangtze River Basin (Sichuan, Chongqing, Hubei, Hunan, Jiangxi and Zhejiang province) and the *P. pashia* planting areas in the subtropical Yunnan–Guizhou Plateau (Yunnan, Guizhou and Guangxi province).

**Figure 8:**
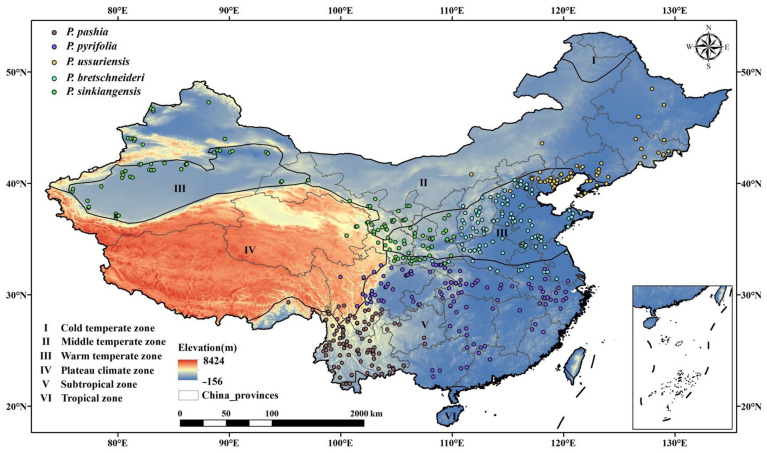
Sample distribution points of five species of *Pyrus* in China. Note: I–VI represents six climatic zones. Five different colored dots represent five species of *Pyrus*.

We conducted a preliminary investigation of species distribution data through online databases, including the Global Biodiversity Information Platform (www.gbif.org, accessed on 3 November 2022), the Chinese National Specimen Resource Platform (www.nsii.org.cn, 28 March 2023), and the China Digital Plant Herbarium (www.cvh.ac.cn, accessed on 3 November 2022) and relative book [48], and 1100 pear distribution points were firstly obtained. Google Earth 7.0 was utilized to find the approximate latitude and longitude, according to the described geographical locations. Then, the R “ENMTooLS” package was used to delete repeated distribution points, to retain one distribution point in one grid [49,50,51] in order to reduce sampling deviation and repetition, and to improve the prediction performance of the model, which could generate a spatial grid corresponding to the resolution of the environmental layer (2.5′ about 22 km^2^). Finally, 510 occurrences were retained for model simulation (Figure 8), including 117 points for *P. bretschneideri*, 55 points for *P. ussuriensis,* 128 points for *P. sinkiangensis*, 98 points for *P. pashia* and 112 points for *P. pyrifolia*.

#### 4.1.2. Geographical and Environmental Data

The grid-scale monthly maximum temperature, monthly minimum temperature and monthly precipitation (spatial resolution is 0.0083333°) under current climate conditions (1990–2020) were from Loess Plateau SubCenter, National Earth System Science Data Center, National Science & Technology Infrastructure of China (http://loess.geodata.cn, accessed on 3 November 2022). This dataset was generated using the delta spatial downscaling method based on the two datasets released by the Climatic Research Unit (CRU) (http://www.cru.uea.ac.uk/, accessed on 12 November 2022) and WorldClim (http://www.worldclim.org, accessed on 12 November 2022) and had been verified by 496 independent meteorological observation points; significant results were achieved [52]. Then, this dataset was processed using the R “dismo” package [53] and “terra” package [54], resulting in 19 climate factors for modeling.

Future bioclimatic variables (2020–2100) were from three General Circulation Models (GCMs) (BCC-CSM2-MR, CanESM5 and CNRM-ESM2-1) in the CMIP6, which have a higher spatial resolution and a more complete description of physical, chemical and biological processes [55] compared to CMIP5. For each GCM, we selected four shared socio-economic pathways (SSPs) (SSP1-2.6, SSP2-4.5, SSP3-7.0 and SSP5-8.5) representing different emission scenarios under future climate conditions [56]. SSPs were combined with the representative concentration pathways (RCPs) [57], providing different pathways of future socioeconomic development and containing possible trends in agriculture and land use. The SSP1-2.6 scenario represented the low-end range of future scenarios measured by its radiative forcing pathway and is predicted to be below 2 °C by 2100. The SSP2-4.5 scenario was considered as a medium stabilization scenario, while the SSP3-7.0 scenario corresponded to the medium- to high-end of the range of future forcing pathways. SSP5-8.5 was the only scenario that stabilized the radiative forcing at 8.5 W/m^2^ in 2100, which was considered to be a high radiative forcing scenario [58]. Finally, the mean value of bioclimatic variables every 20 years from 2020–2100 (2020–2040, 2040–2060, 2060–2080 and 2080–2100) were calculated.

To consider the effects of soil and altitude on planting suitability and elevation, three soil variables (Subsoil Sand Fraction (S_SAND), Subsoil Organic Carbon (S_OC), Subsoil PH (H_2_O) (S_PH_H_2_O)) were added besides bioclimatic variables. The soil data were obtained through the Harmonized World Soil Database version 1.2 (HWSD, http://www.Fao.org, accessed on 21 November 2022) which was constructed by the Food and Agriculture Organization of the United Nations (FAO) and International Institute for Applied Systems Analysis (IIASA) [59]. Elevation data were obtained from the United States Geological Survey (USGS, http://www.usgs.gov, accessed on 21 November 2022).

### 4.2. Model Construction

#### 4.2.1. Model Introduction and Parameter Setting

The model of Maxent (version 3.4.4) [20] was used to build relationships between identified environmental variables (Table 2) and the species distribution data of the selected species to identify suitable areas for each species under current and future climates [24,60]. Compared with other species distribution models, the Maxent model only requires environmental variables and a small amount of point data to obtain better model simulation results for species distribution [21]. To calculate the potential geographic distribution probability of species, we converted the collected geographic coordinate points of the species distribution into the “.csv” format, and unified the layer resolutions of all of the environmental variables used in modeling and the scope of the study area. The number of random background points was set as 10,000. We used 75% of the distribution data for model training and 25% to assess the model’s predictive accuracy [61]. In order to reduce the sampling error caused by randomly splitting the species distribution data into test and training subsets, simulations were repeated 10 times for cross-validation to generate an average result. Jackknife analyses were performed to evaluate the relative importance of each environmental factor for the *Pyrus* distribution modeled. The environmental variables that produced a higher contribution rate and training gain were considered to be more important bioclimatic variables; then, we chose to map the response curves of the dominant environmental variables, and other parameters used default settings.

The logical output result selected by Maxent generated a continuous map with an estimated probability of existence between 0 and 1. The modeled results were divided into five levels by the reclassification tool in ArcGIS 10.2, of which 0–0.05 was considered to be unsuitable, 0.05–0.3 was considered to be lowly unsuitable, 0.3–0.5 was considered to be moderately suitable, 0.5–0.7 was considered to be highly suitable and 0.7–1.0 was considered to be extremely suitable [25,28,62].

#### 4.2.2. Model Evaluation

As identified in other studies, the strong collinearity between any two environmental variables usually results in too high a correlation between them, which affects the simulation results [63,64]. Beginning with 19 bioclimatic variables, the crude annual temperature and precipitation data were firstly deleted. A Pearson correlation analysis of the remaining 17 environmental variables identified the undesirable effects of collinearity on the modeling process and interpretation by SDMToolbox [65] (Figure S1). For each set of significantly cross-correlated variables (Pearson correlation coefficient |r| > 0.8), only one variable was kept for further analysis [66,67]. Ultimately, 12 variables in bold were kept as evaluator variables (Table 2).

The model performance was assessed based on values of the area under the receiver operator curve (AUC) of the receiver operating characteristic (ROC) curve, because it depends on true positive and true negative rates and is not related to the threshold [68]; it is often used in ENMs (environmental niche models) and SDMs (species distribution models) [69,70]. Model performance was classified as failing (0.5–0.6), poor (0.6–0.7), fair (0.7–0.8), good (0.8–0.9) or excellent (0.9–1) [71]. AUC values closer to 1 indicated better-performing models.

### 4.3. Consistency Evaluation of Potential Planting Regions and Actual Main Production Regions

We obtained the current data of the main pear-producing counties (annual output < 1000 tons) and their yield data from 2009 to 2018 using the statistical yearbook of forest products [72]. Excluding the duplication and lack of data in different years, we counted a total of 1027 counties and generated a vector map of the main pear-producing areas in ArcGIS10.2. The different types of suitable distribution maps were superimposed to generate a total distribution map and divided into five levels. Since the suitable planting areas of different species overlap, the output pixel value of the overlapping area was set to the maximum value of the overlapped pixels (different pixel values represent different areas of habitat suitability, ranging from 0 to 1). We superimposed the obtained grading map of the suitable distribution map with the statistical vector map of the main pear-producing counties. If the habitat suitability of the county was greater than 0.5, it was considered that the consistency between the potential planting regions and the actual main production regions of the county was high; otherwise, it was considered that the consistency was low.

## 5. Conclusions

Our research shows that the main pear-producing counties and the potential planting regions simulated by the model have high consistency under the current climate conditions. Based on different emission paths in the future, the area of suitable climate zones of *P. ussuriensis* will decline, while the suitable areas for the other four species will increase from 2020 to 2040, and then decrease until late in the century. As a result of the change in a suitable area of planting *Pyrus*, adaptive management such as cultivating new species and adjusting the planting area are needed to utilize the beneficial effects of climate change or to reduce the adverse effects on *Pyrus*. In addition, the *Pyrus* located in low-altitude and middle-temperate regions will show a more obvious trend of migrating to high-latitude regions in the future, and this changing trend will be more obvious under high-emission scenarios. Therefore, although the carbon dioxide fertilization effect can have positive effects on fruit tree, the potential threat to yield caused by the accelerated reduction in consistency between the suitable planting area and the actual production area of fruit tree under future high emission pathways should be considered. The method can also analyze the impact of climate change on the suitable distribution area and potential yield of other fruit trees combined with other species distribution models.

## Figures and Tables

**Table 1:** Percentage contributions of environmental variables used in study.

Code	Contribution%				
	*P. bretschneideri*	*P. sinkiangensis*	*P. pashia*	*P. pyrifolia*	*P. ussuriensis*
AMT	1.2	1	0.7	0.5	9.4
MDR	1.1	3.9	2.6	1.8	0.1
TS	25.5	9.5	56.6	13.5	37.5
MTCM	33.7	26	3.3	20.7	6.1
AP	0.3	0.2	13.1	53.9	0.1
PS	9.5	12.8	0.2	1	5.8
PWTQ	15.5	3.8	0.1	0.2	25.1
PDQ	0.3	15.1	4.2	3.6	0.5
ALT	11.1	12.3	17.2	2.5	14.7
S_SAND	0.5	1	0.2	0.7	0.6
S_OC	0.4	7.7	0.1	0.7	0
S_PH_H_2_O	1.1	6.6	1.5	1	0.1

**Table 2:** Environmental variables used in the potential distribution modeling of *Pyrus*.

Code	Environment Variables	Units
AMT	Annual mean temperature	℃
MDR	Mean diurnal range	°C
ISO	Isothermality	×100
TS	Temperature seasonality	×100
MTWM	Max. temperature for warmest month	°C
MTCM	Min. temperature for coldest month	°C
TAR	Temperature annual range	℃
MTWTQ	Mean temperature of wettest quarter	°C
MTDQ	Mean temperature of driest quarter	°C
MTWRQ	Mean temperature of warmest quarter	°C
MTCQ	Mean temperature of coldest quarter	°C
AP	Annual precipitation	mm
PWM	Precipitation of wettest month	mm
PDM	Precipitation of driest month	mm
PS	Precipitation seasonality	×100
PWTQ	Precipitation of wettest quarter	mm
PDQ	Precipitation of driest quarter	mm
PWRQ	Precipitation of warmest quarter	mm
PCQ	Precipitation of coldest quarter	mm
Alt	Altitude	m
S_SAND	Subsoil sand fraction	% wt
S_OC	Subsoil organic carbon	% weight
S_PH_H_2_O	Subsoil PH (H_2_O)	−log(H+)

## Data Availability

All relevant data are within the manuscript and its supplementary materials.

## Supplementary Materials

The following supporting information can be downloaded at https://www.mdpi.com/article/10.3390/plants12071559/s1: Figure S1: Correlation analysis table of 17 environmental variables. Figure S2: Distribution map of climatically suitable areas of *P. pashia* under future climatic conditions. Figure S3: Distribution map of climatically suitable areas of *P. ussuriensis* under future climatic conditions. Figure S4: Distribution map of climatically suitable areas of *P. pyrifolia* under future climatic conditions. Figure S5: Distribution map of climatically suitable areas of *P. sinkiangensis* under future climatic conditions.
