# Supplementary material for: Spatial Distribution Characteristics of Suitable Planting Areas for Pyrus Species under Climate Change in China
Source: Plants (Basel). 2023 Apr 5;12(7):1559. doi: 10.3390/plants12071559 (PMC10097120; doi:10.3390/plants12071559)
Supplement: Supplementary file 1 [file plants-12-01559-s001.zip › plants-2317650-supplementary.pdf]

SUPPLEMENTARY INFORMATION:

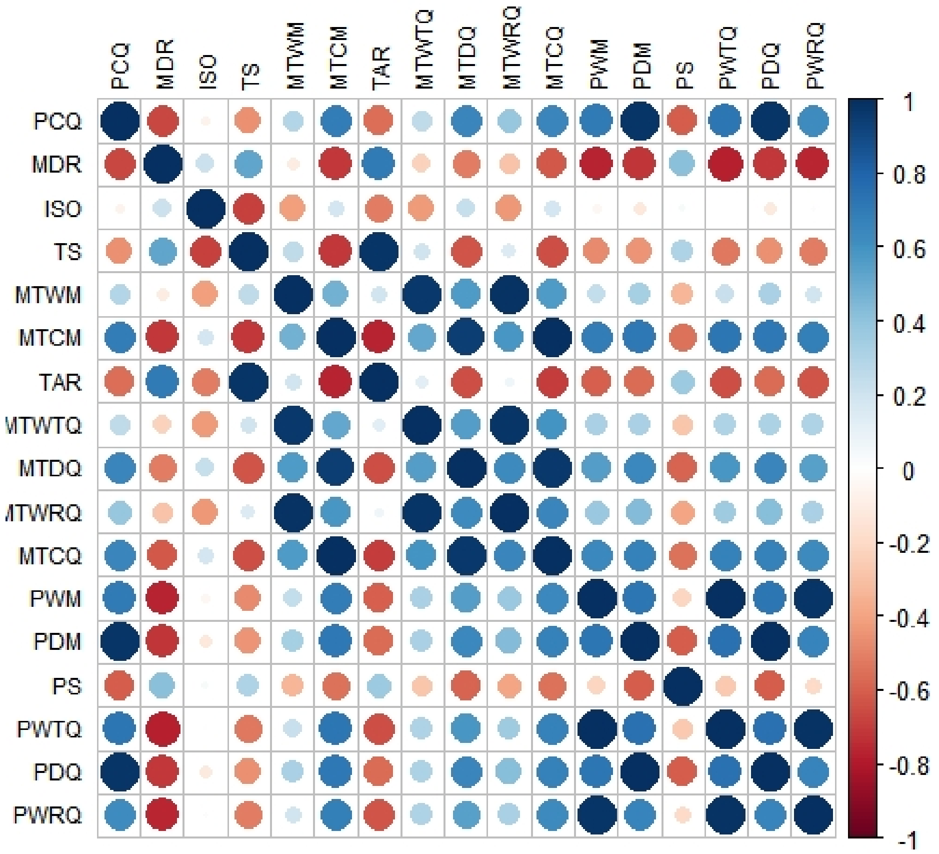

Figure S1. Correlation analysis table of 17 environmental variables

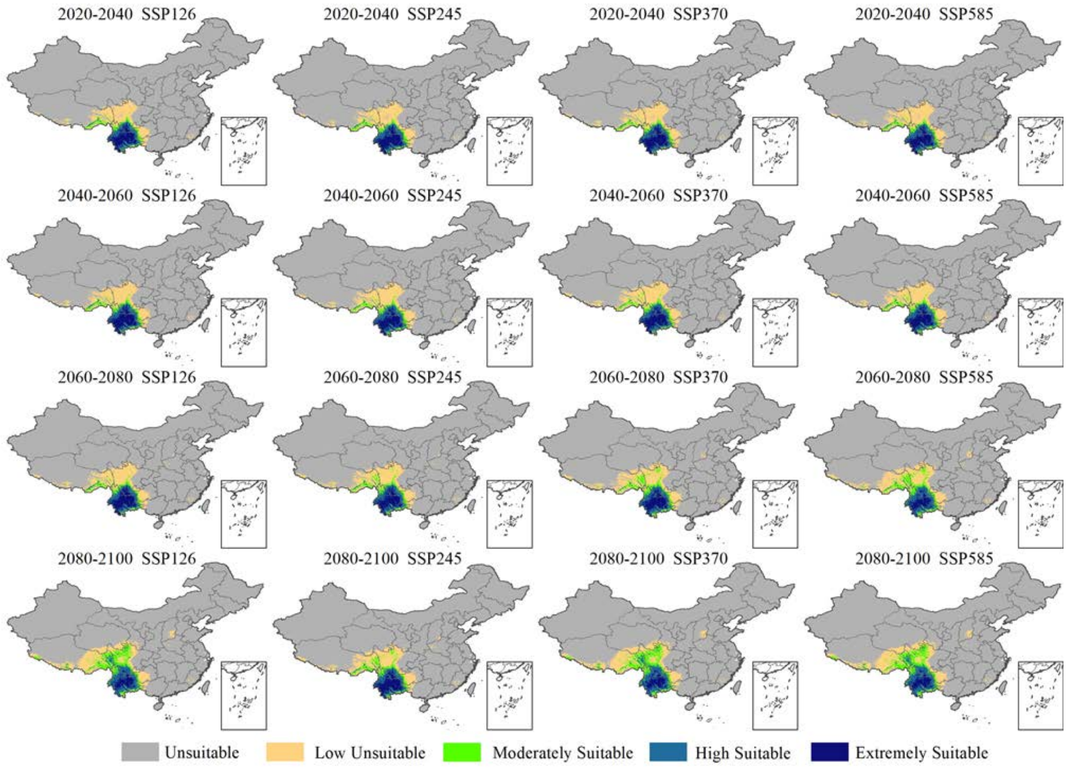

Figure S2. Distribution map of climatically suitable areas of *P. pashia* under future climatic conditions

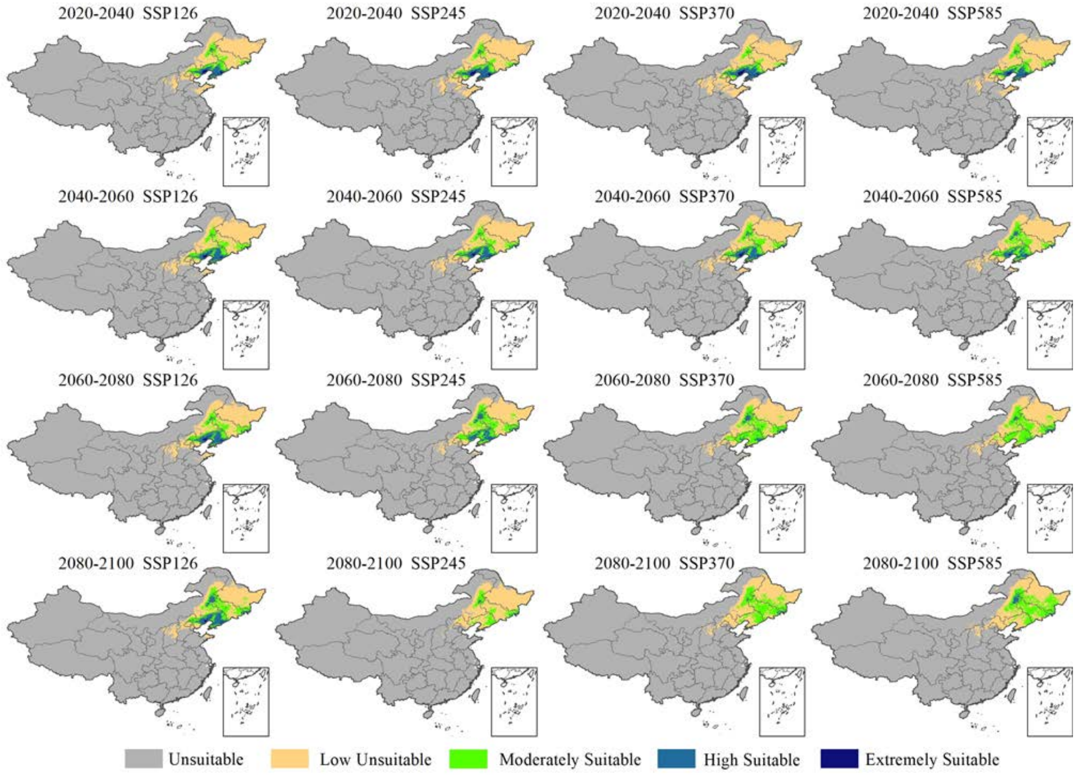

Figurte S3. Distribution map of climatically suitable areas of *P. ussuriensis* under future climatic conditions

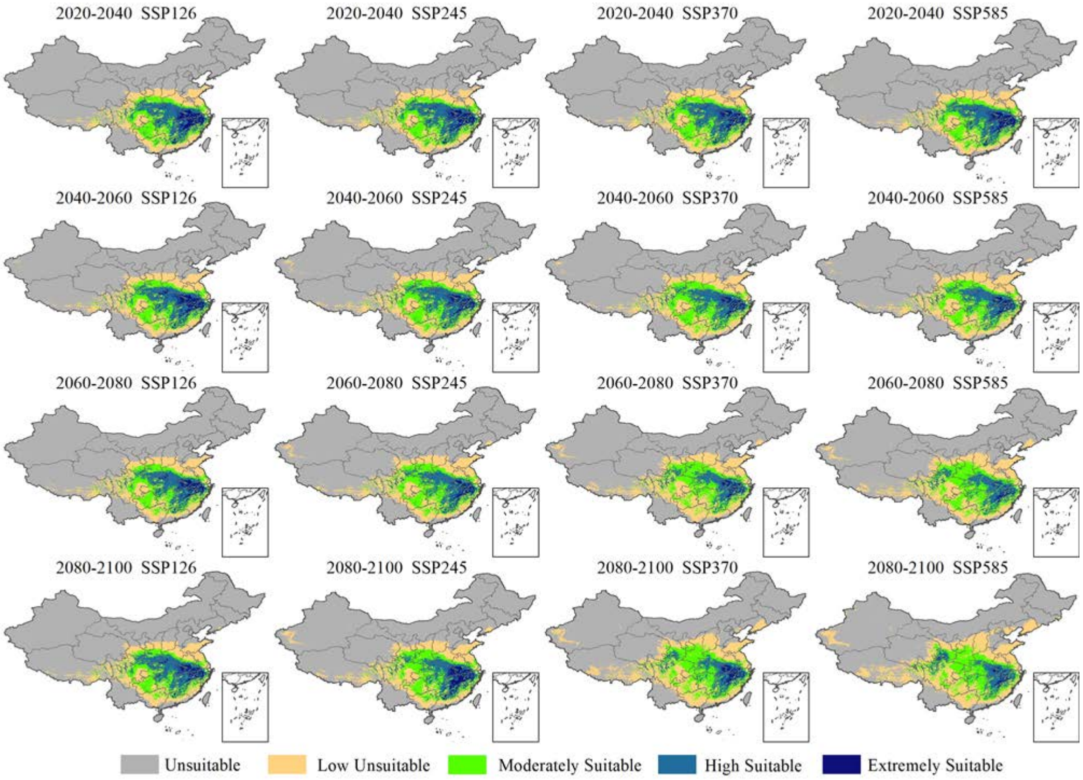

Figure S4. Distribution map of climatically suitable areas of *P. pyrifolia* under future climatic conditions

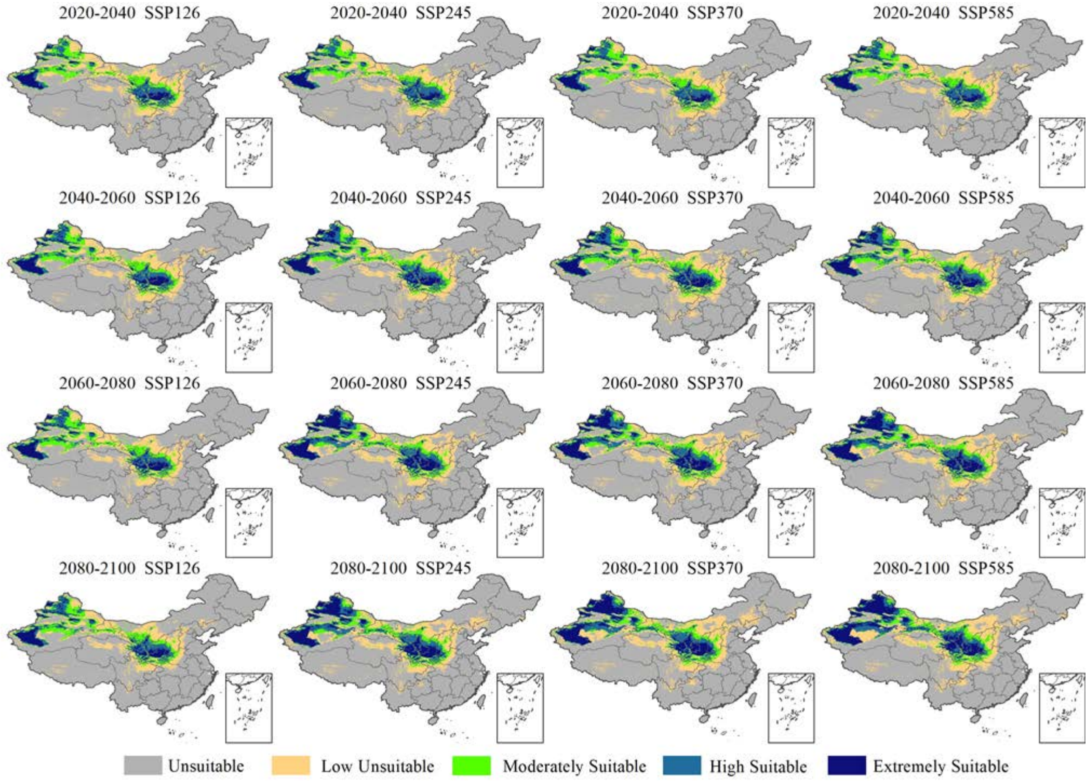

Figure S5. Distribution map of climatically suitable areas of *P. sinkiangensis* under future climatic conditions
